# YAP Translocation Precedes Cytoskeletal Rearrangement in Podocyte Stress Response: A Podometric Investigation of Diabetic Nephropathy

**DOI:** 10.3389/fphys.2021.625762

**Published:** 2021-07-15

**Authors:** Kathryn E. Haley, Mustafa Elshani, In Hwa Um, Cameron Bell, Peter D. Caie, David J. Harrison, Paul A. Reynolds

**Affiliations:** ^1^School of Medicine, University of St Andrews, St Andrews, United Kingdom; ^2^Biomedical Sciences Research Complex (BSRC), University of St Andrews, St Andrews, United Kingdom; ^3^Directorate of Laboratory Medicine, Lothian University Hospitals Trust, Royal Infirmary, Edinburgh, United Kingdom; ^4^Acute Internal Medicine, Queen Elizabeth University Hospital, Glasgow, United Kingdom

**Keywords:** podocytes, diabetic nephropathy, podometrics, YAP, automated image analysis, Hippo signaling

## Abstract

Podocyte loss plays a pivotal role in the pathogenesis of glomerular disease. However, the mechanisms underlying podocyte damage and loss remain poorly understood. Although detachment of viable cells has been documented in experimental Diabetic Nephropathy, correlations between reduced podocyte density and disease severity have not yet been established. YAP, a mechanosensing protein, has recently been shown to correlate with glomerular disease progression, however, the underlying mechanism has yet to be fully elucidated. In this study, we sought to document podocyte density in Diabetic Nephropathy using an amended podometric methodology, and to investigate the interplay between YAP and cytoskeletal integrity during podocyte injury. Podocyte density was quantified using TLE4 and GLEPP1 multiplexed immunofluorescence. Fourteen Diabetic Nephropathy cases were analyzed for both podocyte density and cytoplasmic translocation of YAP via automated image analysis. We demonstrate a significant decrease in podocyte density in Grade III/IV cases (124.5 per 10^6^ μm^3^) relative to Grade I/II cases (226 per 10^6^ μm^3^) (Student’s *t*-test, *p* < 0.001), and further show that YAP translocation precedes cytoskeletal rearrangement following injury. Based on these findings we hypothesize that a significant decrease in podocyte density in late grade Diabetic Nephropathy may be explained by early cytoplasmic translocation of YAP.

## Introduction

Podocytes serve as the final barrier to protein loss *in vivo*. The complex cytoarchitecture of podocytes, consisting of interdigitated foot processes, constitutes the final filtration barrier on the glomerular basement membrane (Pavenstadt et al., 2003). The specialized 40 nm slit diaphragm between podocytes allows for the formation of urine ultrafiltrate while retaining albumin and other essential macromolecules within the blood (Benzing, 2004).

Glomerular diseases including Diabetic Nephropathy and Focal Segmental Glomerulosclerosis (FSGS) are characterized by podocyte loss, resulting in proteinuria (Kihara et al., 1997; Vogelmann et al., 2003; Petermann et al., 2004), defined by loss of more than 3 grams of albumin in the urine per 24 h. This is the primary clinical presentation of glomerular disease (Khanna, 2011). Proteinuria is known to result from disruption of the glomerular filtration barrier due to mislocalization of slit diaphragm proteins such as nephrin, podocin, and CD2AP (Miner, 2002; Zhang et al., 2004; Fukasawa et al., 2009). Moreover, foot process effacement, loss of negative charge at the filtration barrier, impaired endothelial cell integrity, and podocyte hypertrophy can further contribute to impaired glomerular filtration *in vivo* (Saleem, 2015).

It has been shown that reduction in podocyte number is a direct predictor of the extent of segmental scarring that occurs in FSGS (Kriz, 2003; Wharram et al., 2005). Reduction in podocyte number in glomerular disease has been attributed to apoptosis, autophagy, and detachment from the glomerular basement membrane, however, the mechanisms underlying podocyte injury (Kopp et al., 2020) remain largely unknown (Kretzler et al., 1994; Kriz et al., 1994, 1998; Shankland, 2006). While direct relationships between podocyte depletion and progression of glomerulosclerosis have been documented in the PAN-treated rat (Kim et al., 2001), in Diphtheria toxin-induced podocyte injury models (Wharram et al., 2005), and in Angiotensin II-induced injury models (Fukuda et al., 2012), analysis of correlations between podocyte density and disease progression in clinical tissue is limited in Diabetic Nephropathy, where biopsies are not routine clinical practice. However, a recently developed podometric methodology (Venkatareddy et al., 2014) allows for quantification of podocyte density based on staining of a single histologic section. While this technique has been highlighted as a potential tool for monitoring glomerular disease progression (Kikuchi et al., 2015), there remains a need to better understand the mechanisms preceding podocyte detachment *in vivo*.

The pathophysiological steps preceding podocyte detachment have been documented extensively by Kriz et al. (1998), Kriz (2002), and Kriz and LeHir (2005) wherein altered slit diaphragm integrity and cytoskeletal rearrangement have been highlighted as primary effects of podocyte injury. Recently, the mechanosensing protein and Hippo pathway effector, YAP, has emerged as an integral player in mediating the podocyte stress response (Schwartzman et al., 2016; Rinschen et al., 2017; Bonse et al., 2018). Podocyte-specific silencing of YAP *in vivo* results in FSGS (Schwartzman et al., 2016), while podocyte injury stimulates expression of YAP and YAP target genes in a rat model of glomerular disease (Rinschen et al., 2017). The effects of injury on YAP activation in podocytes is substrate-dependent, as a stiff substrate reduced YAP expression in cultured podocytes, whereas a soft substrate stimulated YAP (Rinschen et al., 2017). Recently nuclear YAP was identified as a key regulator of glomerular function (Bonse et al., 2018), where the authors showed that activation of the Hippo pathway leads to a YAP-dependent reduction in podocyte viability.

In light of the emerging role of YAP as a mechanosensing mediator in podocytes, we were interested in whether YAP cytoplasmic translocation in response to injury occurred prior to cytoskeletal rearrangement. Since recent reviews have highlighted the pivotal role of podocytes in the progression of Diabetic Nephropathy (Carney, 2017; Dai et al., 2017), we asked whether we could employ podometric methodology using transducin-like enhancer of split 4 (TLE4) as a nuclear podocyte-specific marker, and glomerular epithelial protein-1 (GLEPP1), a glomerular marker to quantify podocyte density in Diabetic Nephropathy tissue, and further assess the same cohort for expression and localization of YAP.

## Materials and Methods

### Patients

De-identified tissue specimens from 14 adult patients treated at the Edinburgh Royal Infirmary, and diagnosed with Diabetic Nephropathy with no concomitant systemic disease were used for this study. Cases were released under the Ethics of NHS Lothian Bioresource. The estimated glomerular filtration rate (eGFR) was calculated using the following formula: mL/min/m^2^. The degree of hematuria was scored as previously described (Hara et al., 1998). Clinical data for the patients in this cohort is shown in Table 1. Of the patients, 11 were male and 3 were female. For histological grading, 7 were classified as Grade I/II and 7 were Grade III/IV. Proteinuria was reported in clinical charts as 1+, 2+, or 3+ g per day as shown in Supplementary Table 1.

**TABLE 1:** Clinical findings at the time of renal biopsy in Diabetic Nephropathy (*n* = 14).

		Histological grade	
		I/II	III/IV
Sex (M/F)	11 / 3	7 / 0	4 / 3
Age (years)	60.07 ± 2.50	61.86 ± 3.37	58.29 ± 3.84
s-Cr (mg/dL)	2.5 ± 0.56	1.69 ± 0.35	3.44 ± 1.07
eGFR (mL/min/1.73m^2^)	35 ± 5.49	41.57 ± 6.67	27.33 ± 8.51
Proteinuria (g/day)	1.54 ± 0.27	0.96 ± 0.31	2.11 ± 0.35*
Hematuria (scores 0–4)	0.5 ± 0.17	0.33 ± 0.21	0.64 ± 0.28

Data shown are mean ± SD. M, male; F, female; S-Cr, serum-creatinine; eGFR, estimated glomerular filtration rate. Hematuria score: 0 for 0–5 red blood cell/high-powered field (RBC/HPF), 0.5 for 6-10 RBC/HPF, 1.0 for 11-30 RBC/HPF, 2.0 for 31-100 RBC/HPF, 3.0 for > 100 RBC/HPF, 4.0 for numerous RBC/HPF. *p < 0.05 Grade I/II vs. III/IV.

### Renal Histological Findings

Tissue sections used for light microscopy were stained with hematoxylin & eosin (H&E) and periodic acid-Schiff (Brahler et al., 2012). Pathological parameters were analyzed according to the criteria for grading Diabetic Nephropathy as outlined by Tervaert et al. (2010). Histology was assessed for IFTA (0–3), Interstitial inflammation (0–2), Arteriolar hyalinosis (0–2), and Arteriosclerosis (0-2). Severity of disease was graded based on the following criteria noted in the pathology report at the time of biopsy: Grade I: mild or non-specific light microscopy changes and EM-proven GBM thickening. GBM > 395 nm in female and > 430 nm in males. Grade IIa: Mild mesangial expansion in > 25% of the observed mesangium. Grade IIb: Severe mesangial expansion in > 25% of the observed mesangium. Grade III: Nodular sclerosis (Kimmelstiel-Wilson lesion) with at least one convincing lesion noted. Grade IV: Advanced diabetic glomerulosclerosis, with global glomerulosclerosis in > 50% of all glomeruli (Tervaert et al., 2010).

### Primary Antibodies

Mouse monoclonal YAP (1A12) antibody (#12395) was purchased from Cell Signaling Technology (London, United Kingdom). TLE4 (E-10) mouse monoclonal antibody (#sc-365406) was purchased from Insight Biotechnology (Middlesex, United Kingdom) for identification of podocytes. For identification of glomeruli, monoclonal mouse anti-human GLEPP1 (5C11) antibody (#MABS1221) was purchased from Millipore (Hertfordshire, United Kingdom).

### Immunofluorescence of Renal Biopsy Sections-Tyramide Signal Amplification for Visualization of Antibody Binding

Formalin-fixed paraffin embedded (FFPE) needle biopsies from Diabetic Nephropathy patients were cut in 3 μm-thick sections and put onto frosted charged slides. Slides were incubated at 67°C overnight prior to use. FFPE sections were de-paraffinized in fresh xylene for 5 min, thrice. Slides were then rehydrated in 100% ethanol for 2 min, twice, followed by 2 min in 80% ethanol, 2 min in 50% ethanol, and 2 min in running tap water. Antigen retrieval was performed by heating the slides in a pressure cooker containing sodium citrate/citric acid buffer (1.8% 0.1M citric acid, 8.2% 0.1M sodium citrate) in distilled H_2_O. A pressure cooker containing 1L of sodium citrate/citric acid buffer was pre-heated in the microwave at high power for 12 min. Slides were then placed into the pressure cooker and microwaved at high power for 8 min. Once cooled, slides were washed twice for 5 min in PBS-T, followed by a 10-min wash in 3% hydrogen peroxide. Slides were washed twice for 5 min again, and tissue was then outlined with a Dako hydrophobic pen. Blocking was performed with Dako Serum-Free Protein Blocking Solution for 10 min prior to incubation with primary antibodies. Primary antibodies were diluted in Dako antibody diluent and incubated in a humidity chamber for 30 min at room temperature, unless otherwise indicated. Slides were then washed twice for 5 min in PBS-T and incubated in mouse or rabbit HRP-labeled polymer solution for 30 min. Slides were then washed in PBS-T for 5 min, twice. Slides were incubated in cyanine 3, cyanine 5, or FITC Tyramide, respectively (Perkin Elmer, Boston, MA, United States), diluted in TSA diluent (Perkin Elmer, Boston, MA, United States) for 10 min in the dark. Slides were then washed in PBS-T twice for 5 min, and incubated in Hoechst (1:20) for 10 min in the dark. Following two 5 min PBS-T washes, slides were dehydrated in 80% ethanol, air dried, and mounted with ProLong Gold anti-fade mountant (Invitrogen, United Kingdom). Images were captured and analyzed using an automated scanning AQUA microscope (McCabe et al., 2005). Further automated image analysis was performed with Definiens Tissue Studio software.

### Immunofluorescence Multiplexing of Two or More Same Species Antibodies

Multiplexing of two or more same-species antibodies was achieved by heat-induced microwave stripping in sodium-citrate buffer. For this protocol, TSA methodology (McCabe et al., 2005) was adhered to until the TSA fluorophore incubation step. After slides were incubated in the indicated fluorophore, slides were washed twice for 5 min in PBS-T. Sodium citrate buffer was prepared and pre-heated for 12 min at 100% power in a pressure cooker without the rubber seal or pressure top to achieve a lower temperature. Slides were then placed into the pre-heated sodium citrate buffer and heated in the pressure cooker without the rubber seal or steam top for 17 min, on the autodefrost, 750G setting. Slides were then allowed to cool in running tap water for 20 min, and staining with the subsequent primary antibody and corresponding TSA fluorophore was performed.

### Podometric Analysis

The TLE4 method for estimating podocyte density, previously established by Venkatareddy et al. (2014) was used. The methodology detailed above for multiplexing with two primary antibodies was used to co-stain for GLEPP1 and TLE4 via immunofluorescence to measure podocyte cell area density, a calculation previously reported by Yang et al. (2015). Calculation of podocyte nuclear density based on TLE4 immunofluorescence was used as reported by Yang et al. (2015). GLEPP1 positive percentage tuft area was measured based on immunofluorescence, similar to the immunoperoxidase method also reported by Yang et al. (2015). Glomerular tuft volume calculations were made under the assumption that all glomeruli are spherical, as previously published (Hodgin et al., 2015). We adopted the methodologic assumptions used in previously published podometric analysis (Hodgin et al., 2015). A minimum of 6 glomeruli per case were evaluated for podometric analysis. Data acquired from Definiens image analysis was used to calculate podocyte density using the parameters detailed above.

### Definiens Automated Image Analysis

Whole slide images of the immunofluorescence labeled histology sections were acquired for each fluorescence channel (GLEPP1: anti-FITC, TLE4: anti-Cy5, DAPI) with an AQUA PM-2000 platform (HistoRX) microscope at 20× magnification, and were exported without any further processing. Digital image analysis was performed using Definiens Tissue Studio 4.7 software (Definiens AG, Munich). Images acquired, as stated above, were imported into the Tissue Studio software as TIFFs. Tissue area was automatically detected and identified within each image. Definiens’ Composer machine learning technology was utilized to train the algorithm to automatically recognize and segment regions of interest: non-tissue/background regions, or glomeruli, based on GLEPP1 staining. Tissue Studio’s nuclear classification algorithm was used to automatically segment and classify nuclei within the glomeruli. An intensity threshold within the image analysis software was applied to each protein marker to identify cells positive for YAP (threshold intensity: 75), and TLE4 (threshold intensity: 115). Manual correction was used to confirm valid separation of these regions into the correct Regions of Interest (ROI). Co-expression of markers of interest were next used to classify the nuclei for YAP or TLE4 positivity. Tissue Studio’s cell classification algorithm was used to identify number of TLE4 positive cells based on previously set thresholds. Within this population of cells, the nuclear algorithm was used to identify number of cells that were positive for nuclear YAP. These values were then taken as a percentage of total number of TLE4 positive cells per glomerulus.

### Cell Culture

The human immortalized podocyte cell line (Saleem et al., 2002) was obtained as a gift from Prof. Moin Saleem (Bristol, United Kingdom). Proliferating podocytes were cultured at the permissive temperature of 33°C, 5% CO_2_ in RPMI supplemented with 10% Fetal Bovine Serum (FBS), 1% Penicillin-Streptomycin (PS), and 1% Insulin-Transferrin-Selenium (ITS). Podocytes were cultured under differentiation conditions at 37°C, 5% CO_2_ for 12 days in RPMI supplemented with 2% FBS, 1% PS, and 1% ITS. For injury, podocytes were treated with Puromycin Aminonucleoside (PAN) at 25 mg/mL for 24 h, or 30 mM glucose, or hypoxia (0.5% O_2_), followed by 5 day washout of injury condition or return to normoxia.

### Immunocytochemistry

Podocytes were cultured on No.2 glass coverslips in 6 well plates. Cells were washed 2× in PBS, and fixed in 4% paraformaldehyde (PFA) for 10 min at room temperature. Cells were washed for 5 min in PBS, and permeabilized in PBS supplemented with 0.2% Triton X-100 for 5 min, shaking at 15 rpm. Cells were then blocked with 5% Bovine Serum Albumin (BSA), 0.1% Triton X-100 in PBS for 30 min, shaking at 15 rpm. Coverslips were incubated overnight at 4°C in primary antibody: Mouse monoclonal YAP (1A12) antibody (#12395) in 5% BSA, 0.1% Triton X-100 in PBS. Following incubation in primary antibody, coverslips were washed 3× 5 min in PBS, shaking at 15 rpm. Coverslips were incubated in secondary antibody (1:200) Alexa Fluor 568 goat anti-mouse (Life Technologies United Kingdom, Z25006) or in Alexa Fluor 647 Phalloidin (Thermo Fisher Scientific, United Kingdom, A22287) where indicated, in 3% BSA in PBS for 60 min protected from light. Coverslips were then washed 5× 5 min in PBS protected from light, shaking at 15 rpm. Coverslips were mounted on frosted glass slides with Prolong Gold Antifade Reagent with DAPI (Invitrogen United Kingdom, P36931). Slides were imaged with a Leica DM5500 B Microscope.

### Statistical Analysis

Data are presented as mean ± SD. Groups were compared using the unpaired Student’s *t*-test. All analyses were performed using Prism/SPSS software. For correlations with clinical parameters, a linear regression model was used to quantify the relationship between continuous variables.

## Results

### Podometric Analysis Demonstrates Decreased Podocyte Density in Diabetic Nephropathy

**FIGURE 1:**
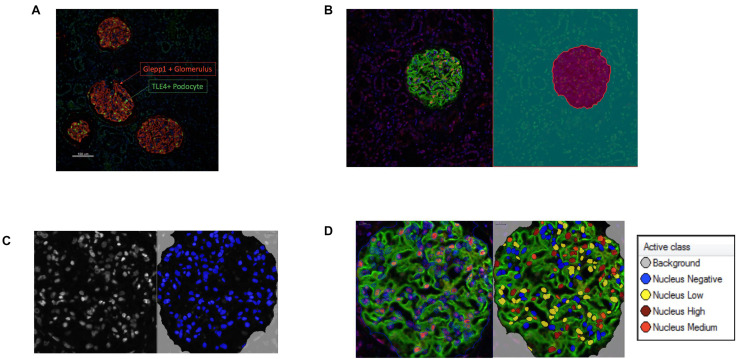
Podometric methodology: Incorporation of automated image analysis **(A)** Representative image of GLEPP1 (red) and TLE4 (green) staining of glomeruli which was analyzed for podocyte density. **(B)** Definiens identification of Region of Interest (ROI) (magenta) based on GLEPP1 staining. **(C)** Hoechst staining is used to segment nuclei within ROI with Definiens. **(D)** Within segmented nuclei, TLE4 expression is used to identify nuclei with low (yellow), medium (orange), and high (red) TLE4 expression levels. Based on a manually determined TLE4 expression threshold, all TLE4-expressing nuclei within the ROI are determined to be podocytes.

Podometric methodology by Hodgin et al. (2015) was amended and used to estimate podocyte density in a Diabetic Nephropathy cohort (*n* = 14). The previously published podometric methodology requires removal of the coverslip for peroxidase immunocytochemistry staining of GLEPP1 following immunofluorescence TLE4 staining. To increase efficiency of the protocol, we employed immunofluorescence multiplexing with two primary antibodies for TLE4 and GLEPP1, allowing the quantification of podocyte number and glomerular tuft area from a single image (Figure 1A). Using automated image analysis, glomeruli were segmented as Regions of Interest (ROIs) based on GLEPP1 staining (Figure 1B), nuclei were segmented based on Hoechst staining (Figure 1C), and TLE4 expression was quantified within each nucleus to identify podocytes (Figure 1D). Since all data was obtained from single images, we were further able to streamline image analysis by using Definiens Tissue Studio software which identified all glomeruli as regions of interest, quantified the number of podocytes within each glomerulus based on a set threshold for TLE4 staining intensity, and generated output of all data required to estimate podocyte density, using a previously reported equation (Venkatareddy et al., 2014).

Diabetic Nephropathy cases were graded based on the pathological classification system published by Tervaert et al. (2010), and stratified into Grade I/II (*n* = 7) vs. Grade III/IV (*n* = 7) before being assessed for significant differences between clinical findings at the time of biopsy (Table 1 and Supplementary Table 1). Of the 14 cases included, the only significant difference between clinical parameters in Grade I/II vs. Grade III/IV was in proteinuria, 0.96 ± 0.31 g/day vs. 2.11 ± 0.35 g/day *(p* < 0.05), respectively (Table 1). Otherwise no significant difference between sex, age, s-Cr, eGFR, or hematuria was observed between the two groups.

**FIGURE 2:**
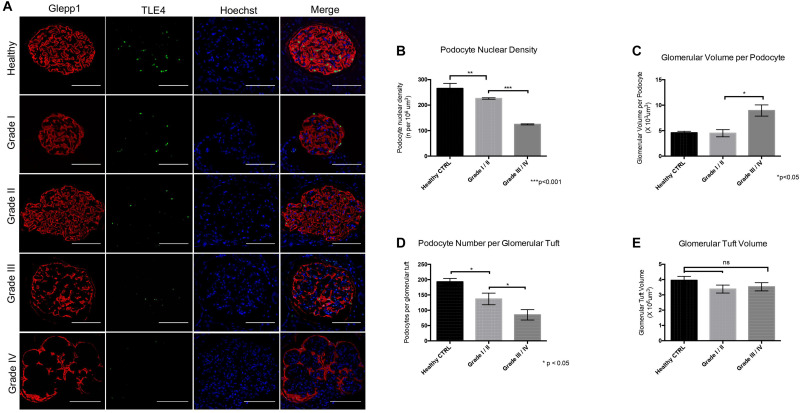
Podometric analysis demonstrates correlation between reduced podocyte density and disease severity in Diabetic Nephropathy **(A)** Representative images of glomeruli from healthy control tissue and Grades I–IV of Diabetic Nephropathy cohort. GLEPP1 (red) was used as a glomerular marker, TLE4 (green) was used as a nuclear podocyte marker, Hoechst (blue) was used as a nuclear marker. **(B)** Podocyte nuclear density (n per 10^6^um^3^) in healthy vs. Grade I/II vs. Grade III/IV of Diabetic Nephropathy (**p < 0.01 and ***p < 0.001). **(C)** Glomerular volume per podocyte (×10^3^um^3^) in Grade I/II vs. Grade III/IV in Diabetic Nephropathy (*p < 0.05). **(D)** Podocyte number per glomerular tuft in Grade I/II vs. Grade III/IV in Diabetic Nephropathy (*p < 0.05). **(E)** Glomerular tuft volume (x10^6^ um^3^) in Grade I/II vs. Grade III/IV in Diabetic Nephropathy (ns, not significant).

Immunofluorescence labeling of glomeruli with GLEPP1 as a glomerular marker and TLE4 as a podocyte nuclear marker revealed a decrease in podocyte density in all grades of Diabetic Nephropathy cases relative to healthy control tissue (Figures 2A,B). The lowest podocyte density was observed in Grade 4 Diabetic Nephropathy cases, with podocyte density gradually decreasing from Grade 1 through Grade 4 as disease severity increased (Figure 2A). Podocyte nuclear density decreased by 55% when comparing Grade I/II to Grade III/IV, with a mean podocyte density of 226 podocytes per 10^6^ μm^3^ glomerular volume in Grade I/II vs. 124.5 per 10^6^ μm^3^ in Grade III/IV (*p* < 0.001) (Figure 2B). Glomerular volume per podocyte increased almost two-fold between Grade I/II and Grade III/IV, with a mean glomerular volume per podocyte of 4.5 × 10^3^ μm^3^ in Grade I/II cases vs. 8.96 × 10^3^ μm^3^ in Grade III/IV cases (*p* < 0.05) (Figure 2C). Consistent with the decrease in podocyte nuclear density observed, podocyte number per glomerular tuft significantly decreased from Grade I/II to Grade III/IV cases, with an average of 137 ± 19 podocytes per tuft in Grade I/II cases as compared to an average of 85 ± 17 podocytes per tuft in Grade III/IV cases (*p* < 0.05) (Figure 2D). However, when comparing glomerular tuft volume between Grade I/II and Grade III/IV cases, no significant difference was observed (Figure 2E). Collectively, the podometric analysis of this cohort demonstrates that podocyte density significantly decreases in late stage Diabetic Nephropathy, consistent with other previously published findings (Pagtalunan et al., 1997; Meyer et al., 1999; Steffes et al., 2001; White et al., 2002).

### Loss of Nuclear YAP in Late Grade Diabetic Nephropathy

**FIGURE 3:**
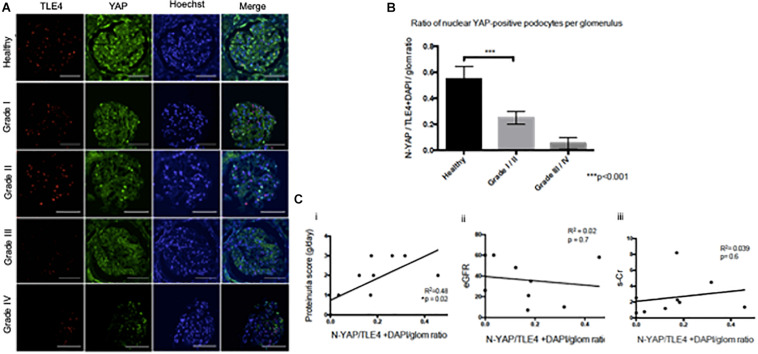
Loss of nuclear YAP correlates with disease severity in Diabetic Nephropathy **(A)** Representative images of glomeruli from healthy renal cortical tissue and from Grade I–IV Diabetic Nephropathy cases stained with TLE4 (red) as a podocyte nuclear marker, YAP (green) and Hoechst (blue) as a nuclear marker. Magnification: 20×. Scale bar = 100 μm. **(B)** Ratio of YAP-positive nuclei (N-YAP/TE4+DAPI/glom) based on histological classification of disease (Grade I-II vs. Grade III-IV). Values expressed as mean ± SD. ***p < 0.001, Student’s *t*-test, unpaired. **(C)** Assessment of the relationship between clinical parameters (proteinuria, eGFR, serum creatinine, hematuria) and ratio of YAP-positive podocyte nuclei. (i) Proteinuria v. N-YAP/TLE4+DAPI/glom ratio, linear regression, *p = 0.024 (ii) Serum creatinine (s-Cr) v. N-YAP/TLE4+DAPI/glom ratio, *p* = 0.6 (iii) Estimated glomerular filtration rate (eGFR) v. N-YAP/TLE4+DAPI/glom ratio, *p* = 0.7. Correlation between variables was evaluated by least-squares linear regression.

While both cytoplasmic and nuclear YAP expression was observed in healthy tissue, we observed cytoplasmic translocation of YAP in late grade Diabetic Nephropathy (Figures 3A,B). We then calculated nuclear YAP per podocyte per glomerulus, a metric comparable to that previously used to calculate podocyte expression of nuclear dendrin (Asanuma et al., 2011). This analysis demonstrated a significant decrease in nuclear YAP in Grade I/II Diabetic Nephropathy relative to healthy tissue (Figure 3B). A correlation was observed between podocyte nuclear YAP expression and proteinuria that achieved significance at *p* = 0.02, but the evidence that there is a direct association between podocyte nuclear YAP expression and proteinuria is not strong (*r*^2^ = 0.48), while other clinical parameters including eGFR and serum creatinine showed no significant correlation with nuclear YAP expression (Figure 3C).

### Recovery of YAP Following Exposure to High Glucose and Hypoxia *in vitro*

**FIGURE 4:**
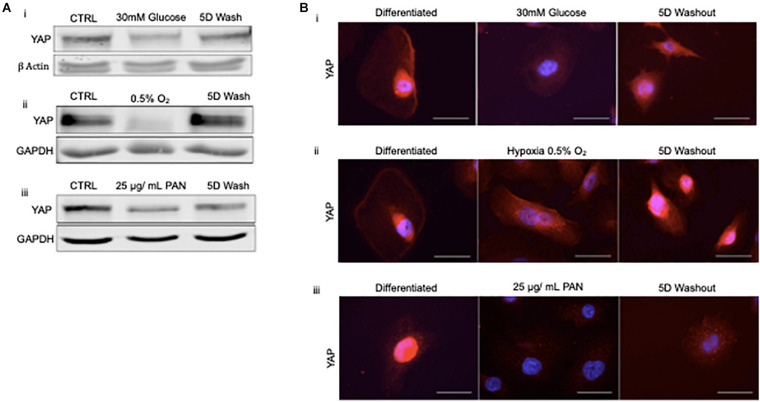
Podocyte stress response induces reversible cytoplasmic translocation of YAP *in vitro* **(A)** Western blots for YAP comparing differentiated control podocytes, injured podocytes (i) 30 mM glucose, (ii) hypoxia (0.5% O_2_), (iii) 25 μg/mL PAN, and 5 day washout of injury condition or return to normoxia. β-Actin and GAPDH were used as loading controls. **(B)** Representative immunofluorescence images of YAP expression (red) in podocytes treated with (i) 30mM glucose, (ii) hypoxia (0.5% O_2_), and (iii) 25 μg/mL PAN compared to differentiated control and 5 day washout of injury condition. DAPI (blue) detects nuclei. Magnification, 40×. Scale bar = 50 μm.

To address whether podocyte injury was reversible, we then assessed the expression and localization of YAP in human podocytes *in vitro*. Total YAP protein expression decreased following injury with 30 mM glucose, hypoxia, and 25 μg/ml PAN (Figure 4A). YAP expression was partially recovered in podocytes treated with high glucose following 5 day washout, whereas cells exposed to hypoxia completely recovered expression following washout and return to normoxia for 5 days. Endogenous nuclear YAP was present in healthy differentiated podocytes *in vitro* based on immunofluorescence and confocal microscopy imaging (Figure 4B). The induction of stress (by 30mM glucose, hypoxia, or PAN treatment) resulted in reduced endogenous nuclear YAP expression, which was partially recovered following 5 day washout of both 30mM glucose and hypoxia, but not PAN (Figure 4B).

### YAP Translocation Precedes Cytoskeletal Rearrangement During Podocyte Stress Response

**FIGURE 5:**
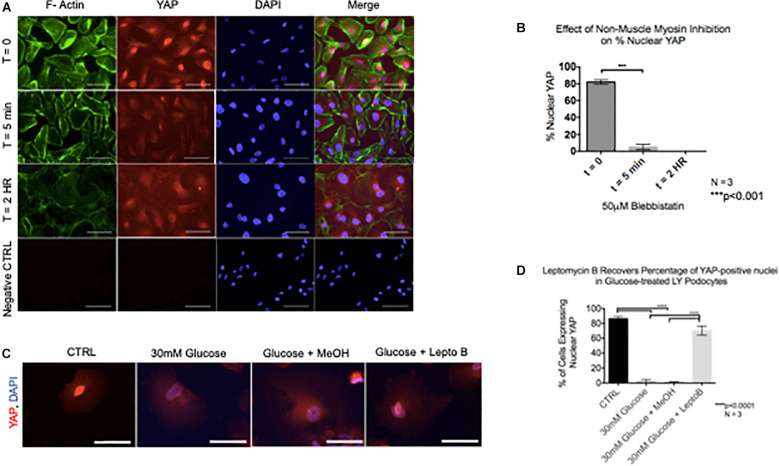
YAP translocation precedes cytoskeletal rearrangement during podocyte stress response **(A)** Immunofluorescence staining demonstrates F-Actin (green) and YAP (red) localization in podocytes treated with non-muscle myosin inhibitor, Blebbistatin. DAPI (blue) detects nuclei. **(B)** Quantification of percentage of nuclear YAP positive cells following Blebbistatin treatment. 100 cells per timepoint per experiment were assessed for YAP localization, *N* = 3. ***p < 0.001, one-way ANOVA, multiple comparisons test, data presented as mean ± SD. **(C)** Immunofluorescence staining demonstrates YAP (red) and DAPI (blue) staining in podocytes treated with glucose, glucose + vehicle, and glucose + Leptomycin B. Magnification, 40×. Scale bar = 50 μm. **(D)** Quantification of cells expressing nuclear YAP in immunofluorescence images of all conditions. One way ANOVA, multiple comparisons test, data presented as mean ± SD, ****p < 0.0001, and *N* = 3.

Given that cytoskeletal rearrangement is known to be a pivotal step in podocyte foot process effacement and subsequent detachment, we next asked whether YAP translocation during podocyte stress-response precedes cytoskeletal rearrangement. Interestingly, following 5 min of treatment with Blebbistatin, a well characterized specific inhibitor of non-muscle myosin, there was a significant decrease in the percentage of cells with nuclear YAP (82.1% ± 1.5% vs. 5.2% ± 1.8%) (Figures 5A,B). Following 2 h of Blebbistatin treatment, YAP was localized exclusively in the cytoplasm, and cytoskeletal structure was non-striated and disorganized (Figure 5A). Having identified that cytoplasmic translocation of YAP during non-muscle myosin inhibition occurs prior to cytoskeletal rearrangement, we then asked whether inhibition of nuclear export would retain the nuclear YAP phenotype during podocyte stress-response. To test this, human podocytes were cultured in glucose in addition to Leptomycin B, a known inhibitor of nuclear export which targets CRM1, a receptor for the nuclear export signal of proteins. While glucose treatment resulted in cytoplasmic translocation of YAP as previously observed, co-culture with glucose and Leptomycin B resulted in retention of nuclear YAP, comparable to the control phenotype (86.3% ± 1.8% vs. 70.3% ± 3.6%) (Figures 5C,D).

## Discussion

Progression of glomerular disease resulting from diabetes has been characterized by decreased podocyte density (Pagtalunan et al., 1997; Steffes et al., 2001) which can result from podocyte apoptosis or detachment of viable podocytes into the urine (Petermann et al., 2004). Podometric methodology has been previously used to analyze podocyte density in aging glomeruli (Hodgin et al., 2015) as well as in transplant glomeruli (Yang et al., 2015), and has been highlighted as a potential tool for glomerular disease management (Kikuchi et al., 2015). Here, we present an amended and streamlined methodology for podometric staining and image analysis by multiplexing TLE4 and GLEPP1 primary antibodies for immunofluorescence, and utilizing image analysis with Definiens software to allow for automated detection and quantification of glomerular metrics and podocyte number across a cohort of tissue slides.

The podometric methodology developed herein offers two advantages over the existing methodology. First, by multiplexing primary antibodies, immunofluorescence can be performed for both TLE4 and GLEPP1 simultaneously on the same tissue section, without requiring removal of a coverslip. By streamlining the protocol, we make its use in a clinical setting a feasible goal. The second advantage is the incorporation of automated image analysis for quantification of the variables required for calculation of podocyte density. Incorporation of automated image analysis allows for automated batch analysis of tissue sections, and increases the efficiency of the methodology, making it more suitable as a prognostic tool in a clinical setting. We stratified the cohort into early grade vs. late grade Diabetic Nephropathy as defined by the pathological classification detailed by Tervaert et al. (2010). We demonstrate decreased podocyte nuclear density in Grades III and IV relative to Grades I and II, consistent with previous findings of podocyte loss in both type I and type II diabetes (Pagtalunan et al., 1997; Steffes et al., 2001).

Since recent publications (Schwartzman et al., 2016; Rinschen et al., 2017; Bonse et al., 2018) have identified YAP as an integral player in podocyte stress response through both mechanosensing and Hippo pathway signaling, we were interested in assessing YAP localization in human Diabetic Nephropathy cases through machine learning image analysis, and further investigating the sequence of events resulting in YAP translocation and cytoskeletal rearrangement during podocyte stress response *in vitro*. Although we were limited to a small cohort, we observed loss of nuclear YAP in podocytes in late grade Diabetic Nephropathy, most notably in Grade IV cases, and we observed nuclear YAP in healthy differentiated human podocytes *in vitro*. Given previously reported observations that YAP localization was specific to substrate stiffness *in vitro*, and that substrate stiffness is known to affect cytoskeletal integrity, we asked whether YAP translocation during stress response preceded cytoskeletal rearrangement, as this sequence of events would have important implications for developing strategic therapeutic approaches. Interestingly, we observed that Blebbistatin treatment induced cytoplasmic translocation of YAP prior to cytoskeletal rearrangement, suggesting that YAP may play a role in regulating cytoskeletal dynamics in human podocytes. Collectively, our observations in clinical tissue and *in vitro* warrant further investigation of the interplay between YAP and cytoskeletal integrity in maintaining podocyte viability.

To conclude, while biopsy of Diabetic Nephropathy cases is not common clinical practice and thus limits the cohort available for future studies, the amended podometric methodology presented herein demonstrates technical improvement for obtaining podometric data, and a streamlined process for quantitative image analysis which may be used in a clinical setting as a diagnostic tool for other glomerular diseases where obtaining a biopsy is standard clinical practice, such as FSGS. Additionally, we document early translocation of YAP which precedes cytoskeletal rearrangement during stress-response, suggesting that YAP may serve as a pivotal target early on in glomerular disease onset.

## Data Availability Statement

The original contributions presented in the study are included in the article/Supplementary Material, further inquiries can be directed to the corresponding author.

## Ethics Statement

The studies involving human participants were reviewed and approved by University of St Andrews (UTREC). The patients/participants provided their written informed consent to participate in this study.

## Author Contributions

KH, ME, DH, and PR contributed to the conception and design of research, and edited and revised the manuscript. KH and CB performed the experiments. KH, ME, and IU analyzed the data. KH, ME, PC, DH, and PR interpreted the results of the experiments. KH prepared the figures and drafted the manuscript. KH, ME, CB, IU, PC, DH, and PR approved the final version of the manuscript. All authors contributed to the article and approved the submitted version.

## Conflict of Interest

The authors declare that the research was conducted in the absence of any commercial or financial relationships that could be construed as a potential conflict of interest.

## Supplementary Material

Supplementary Table 1 Clinical and laboratory findings at the time of biopsy for each case. Unk, unknown.

## References

[B1] Asanuma K. Akiba-Takagi M. Kodama F. Asao R. Nagai Y. Lydia A. (2011). Dendrin location in podocytes is associated with disease progression in animal and human glomerulopathy. *Am. J. Nephrol.* 33 537–549. 10.1159/000327995 21606645

[B2] Benzing T. (2004). Signaling at the slit diaphragm. *J. Am. Soc. Nephrol.* 15 1382–1391. 10.1097/01.asn.0000130167.30769.55 15153549

[B3] Bonse J. Wennmann D. O. Kremerskothen J. Weide T. Michgehl U. Pavenstadt H. (2018). Nuclear YAP localization as a key regulator of podocyte function. *Cell Death Dis.* 9:850. 10.1038/s41419-018-0878-1 PMC6113334 30154411

[B4] Brahler S. Ising C. Hagmann H. Rasmus M. Hoehne M. Kurschat C. (2012). Intrinsic proinflammatory signaling in podocytes contributes to podocyte damage and prolonged proteinuria. *Am. J. Physiol. Renal Physiol.* 303 F1473–F1485. 22975019 10.1152/ajprenal.00031.2012

[B5] Carney E. F. (2017). Diabetic nephropathy: restoring podocyte proteostasis in DN. *Nat. Rev. Nephrol.* 13:514. 10.1038/nrneph.2017.111 28736434

[B6] Dai H. Liu Q. Liu B. (2017). Research progress on mechanism of podocyte depletion in diabetic nephropathy. *J. Diabetes Res.* 2017:2615286. 10.1155/2017/2615286 PMC5534294 28791309

[B7] Fukasawa H. Bornheimer S. Kudlicka K. Farquhar M. G. (2009). Slit diaphragms contain tight junction proteins. *J. Am. Soc. Nephrol.* 20 1491–1503. 10.1681/asn.2008101117 19478094 PMC2709684

[B8] Fukuda A. Wickman L. T. Venkatareddy M. P. Sato Y. Chowdhury M. A. Wang S. Q. (2012). Angiotensin II-dependent persistent podocyte loss from destabilized glomeruli causes progression of end stage kidney disease. *Kidney Int.* 81 40–55. 10.1038/ki.2011.306 21937979 PMC3739490

[B9] Hara M. Yanagihara T. Takada T. Itoh M. Matsuno M. Yamamoto T. (1998). Urinary excretion of podocytes reflects disease activity in children with glomerulonephritis. *Am. J. Nephrol.* 18 35–41. 10.1159/000013302 9481437

[B10] Hodgin J. B. Bitzer M. Wickman L. Afshinnia F. Wang S. Q. O’Connor C. (2015). Glomerular aging and focal global glomerulosclerosis: a podometric perspective. *J. Am. Soc. Nephrol.* 26 3162–3178. 10.1681/asn.2014080752 26038526 PMC4657829

[B11] Khanna R. (2011). Clinical presentation & management of glomerular diseases: hematuria, nephritic & nephrotic syndrome. *Mo. Med.* 108 33–36. 21462608 PMC6188440

[B12] Kihara I. Tsuchida S. Yaoita E. Yamamoto T. Hara M. Yanagihara T. (1997). Podocyte detachment and epithelial cell reaction in focal segmental glomerulosclerosis with cellular variants. *Kidney Int. Suppl.* 63 S171–S176. 9407451

[B13] Kikuchi M. Wickman L. Hodgin J. B. Wiggins R. C. (2015). Podometrics as a potential clinical tool for glomerular disease management. *Semin. Nephrol.* 35 245–255. 10.1016/j.semnephrol.2015.04.004 26215862 PMC4518207

[B14] Kim Y. H. Goyal M. Kurnit D. Wharram B. Wiggins J. Holzman L. (2001). Podocyte depletion and glomerulosclerosis have a direct relationship in the PAN-treated rat. *Kidney Int.* 60 957–968. 10.1046/j.1523-1755.2001.060003957.x 11532090

[B15] Kopp J. B. Anders H. J. Susztak K. Podesta M. A. Remuzzi G. Hildebrandt F. (2020). Podocytopathies. *Nat. Rev. Dis. Primers* 6:68. 10.1038/s41572-020-0196-7 PMC8162925 32792490

[B16] Kretzler M. Koeppen-Hagemann I. Kriz W. (1994). Podocyte damage is a critical step in the development of glomerulosclerosis in the uninephrectomised-desoxycorticosterone hypertensive rat. *Virchows Arch.* 425 181–193. 7952502 10.1007/BF00230355

[B17] Kriz W. (2002). Podocyte is the major culprit accounting for the progression of chronic renal disease. *Microsc. Res. Tech.* 57 189–195. 10.1002/jemt.10072 12012382

[B18] Kriz W. (2003). Progression of chronic renal failure in focal segmental glomerulosclerosis: consequence of podocyte damage or of tubulointerstitial fibrosis? *Pediatr. Nephrol.* 18 617–622. 10.1007/s00467-003-1172-7 12879860

[B19] Kriz W. Elger M. Nagata M. Kretzler M. Uiker S. Koeppen-Hageman I. (1994). The role of podocytes in the development of glomerular sclerosis. *Kidney Int. Suppl.* 45 S64–S72. 8158902

[B20] Kriz W. Gretz N. Lemley K. V. (1998). Progression of glomerular diseases: is the podocyte the culprit? *Kidney Int.* 54 687–697. 10.1046/j.1523-1755.1998.00044.x 9734594

[B21] Kriz W. LeHir M. (2005). Pathways to nephron loss starting from glomerular diseases-insights from animal models. *Kidney Int.* 67 404–419. 10.1111/j.1523-1755.2005.67097.x 15673288

[B22] McCabe A. Dolled-Filhart M. Camp R. L. Rimm D. L. (2005). Automated quantitative analysis (AQUA) of in situ protein expression, antibody concentration, and prognosis. *J. Natl. Cancer Inst.* 97 1808–1815. 10.1093/jnci/dji427 16368942

[B23] Meyer T. W. Bennett P. H. Nelson R. G. (1999). Podocyte number predicts long-term urinary albumin excretion in Pima Indians with Type II diabetes and microalbuminuria. *Diabetologia* 42 1341–1344. 10.1007/s001250051447 10550418

[B24] Miner J. H. (2002). Focusing on the glomerular slit diaphragm: podocin enters the picture. *Am. J. Pathol.* 160 3–5. 10.1016/s0002-9440(10)64341-6 11786391 PMC1867141

[B25] Pagtalunan M. E. Miller P. L. Jumping-Eagle S. Nelson R. G. Myers B. D. Rennke H. G. (1997). Podocyte loss and progressive glomerular injury in type II diabetes. *J. Clin. Invest.* 99 342–348. 10.1172/jci119163 9006003 PMC507802

[B26] Pavenstadt H. Kriz W. Kretzler M. (2003). Cell biology of the glomerular podocyte. *Physiol. Rev.* 83 253–307. 10.1152/physrev.00020.2002 12506131

[B27] Petermann A. T. Pippin J. Krofft R. Blonski M. Griffin S. Durvasula R. (2004). Viable podocytes detach in experimental diabetic nephropathy: potential mechanism underlying glomerulosclerosis. *Nephron Exp. Nephrol.* 98 e114–e123. 15627794 10.1159/000081555

[B28] Rinschen M. M. Grahammer F. Hoppe A. K. Kohli P. Hagmann H. Kretz O. (2017). YAP-mediated mechanotransduction determines the podocyte’s response to damage. *Sci. Signal.* 10:eaaf8165. 10.1126/scisignal.aaf8165 28400537

[B29] Saleem M. A. (2015). One hundred ways to kill a podocyte. *Nephrol. Dial. Transplant.* 30 1266–1271. 10.1093/ndt/gfu363 25637640

[B30] Saleem M. A. O’Hare M. J. Reiser J. Coward R. J. Inward C. D. Farren T. (2002). A conditionally immortalized human podocyte cell line demonstrating nephrin and podocin expression. *J. Am. Soc. Nephrol.* 13 630–638. 10.1681/asn.v133630 11856766

[B31] Schwartzman M. Reginensi A. Wong J. S. Basgen J. M. Meliambro K. Nicholas S. B. (2016). Podocyte-specific deletion of yes-associated protein causes FSGS and progressive renal failure. *J. Am. Soc. Nephrol.* 27 216–226. 10.1681/asn.2014090916 26015453 PMC4696566

[B32] Shankland S. J. (2006). The podocyte’s response to injury: role in proteinuria and glomerulosclerosis. *Kidney Int.* 69 2131–2147. 10.1038/sj.ki.5000410 16688120

[B33] Steffes M. W. Schmidt D. McCrery R. Basgen J. M., and International Diabetic Nephropathy Study Group (2001). Glomerular cell number in normal subjects and in type 1 diabetic patients. *Kidney Int.* 59 2104–2113. 10.1046/j.1523-1755.2001.0590062104.x 11380812

[B34] Tervaert T. W. Mooyaart A. L. Amann K. Cohen A. H. Cook H. T. Drachenberg C. B. (2010). Pathologic classification of diabetic nephropathy. *J. Am. Soc. Nephrol.* 21 556–563. 20167701 10.1681/ASN.2010010010

[B35] Venkatareddy M. Wang S. Yang Y. Patel S. Wickman L. Nishizono R. (2014). Estimating podocyte number and density using a single histologic section. *J. Am. Soc. Nephrol.* 25 1118–1129. 10.1681/asn.2013080859 24357669 PMC4005315

[B36] Vogelmann S. U. Nelson W. J. Myers B. D. Lemley K. V. (2003). Urinary excretion of viable podocytes in health and renal disease. *Am. J. Physiol. Renal Physiol.* 285 F40–F48. 12631553 10.1152/ajprenal.00404.2002 PMC3368602

[B37] Wharram B. L. Goyal M. Wiggins J. E. Sanden S. K. Hussain S. Filipiak W. E. (2005). Podocyte depletion causes glomerulosclerosis: diphtheria toxin-induced podocyte depletion in rats expressing human diphtheria toxin receptor transgene. *J. Am. Soc. Nephrol.* 16 2941–2952. 10.1681/asn.2005010055 16107576

[B38] White K. E. Bilous R. W. Marshall S. M. El Nahas M. Remuzzi G. Piras G. (2002). Podocyte number in normotensive type 1 diabetic patients with albuminuria. *Diabetes* 51 3083–3089. 10.2337/diabetes.51.10.3083 12351451

[B39] Yang Y. Hodgin J. B. Afshinnia F. Wang S. Q. Wickman L. Chowdhury M. (2015). The two kidney to one kidney transition and transplant glomerulopathy: a podocyte perspective. *J. Am. Soc. Nephrol.* 26 1450–1465. 10.1681/asn.2014030287 25388223 PMC4446871

[B40] Zhang S. Y. Marlier A. Gribouval O. Gilbert T. Heidet L. Antignac C. (2004). In vivo expression of podocyte slit diaphragm-associated proteins in nephrotic patients with NPHS2 mutation. *Kidney Int.* 66 945–954. 10.1111/j.1523-1755.2004.00840.x 15327385

