# Supplementary material for: YAP Translocation Precedes Cytoskeletal Rearrangement in Podocyte Stress Response: A Podometric Investigation of Diabetic Nephropathy
Source: Front Physiol. 2021 Jul 15;12:625762. doi: 10.3389/fphys.2021.625762 (PMC8320019; doi:10.3389/fphys.2021.625762)
Supplement: Supplementary Table 1 — Clinical and laboratory findings at the time of biopsy for each case. Unk, unknown. [file Table_1.pdf]

Sample ID	Grade (I-IV)	Age (years)	Sex (M/F)	s-Cr (mg/dL)	Proteinuria (g/Day)	Hematuria (0-4)	eGFR (mL/min/1.73m^2)
1	2a	75	M	2.87	0.78	1	22
2	3	62	M	3.18	0.78	0.5	20
3	2a	58	M	0.8	2	0	60
4	3	51	F	1.19	2	0	48
5	1	66	M	1.95	2	0	35
6	2a	51	M	0.63	0	0	60
7	3	45	M	1.35	2	1	58
8	3	54	M	8.19	1	2	7
9	2a	51	M	0.77	1	1	60
10	4	61	F	4.48	3	0	10
11	4	77	F	2.26	3	1	21
12	2a	67	M	2.31	1	0	28
13	2b	65	M	2.52	0	UNK	26
14	3	58	M	UNK	3	0	UNK

Table S1. Clinical and laboratory findings at the time of biopsy for each case. UNK= Unknown
